# Effective chemotherapy and targeted therapy supplemented with stereotactic radiotherapy of a patient with metastatic colon cancer following renal transplantation: a case report

**DOI:** 10.1186/s13256-021-02702-y

**Published:** 2021-03-20

**Authors:** Szabolcs Bellyei, Árpád Boronkai, Eva Pozsgai, Dávid Fodor, László Mangel

**Affiliations:** 1 Department of Oncotherapy, Clinical Center, University of Pécs, Édesanyák Street 10, 7624 Pécs, Hungary; 2 Department of Public Health, Medical School, University of Pécs, Szigeti Street 12, 7624 Pécs, Hungary; 3 Institute of Primary Health Care, Medical School, University of Pécs, Rákóczi Street 2, 7623 Pécs, Hungary

**Keywords:** Colorectal cancer, Kidney transplantation, Stereotactic body irradiation, Palliative chemotherapy, Targeted treatment

## Abstract

**Background:**

Previous studies have shown that patients who underwent renal transplantation were at a greater risk of developing malignancies. Due to advances in effective surgical techniques and immunosuppressive therapies, organ recipients live longer. Yet, there is insufficient information about the recommended type of therapy for colorectal cancer patients following transplantation. We describe the oncological treatment of a patient with renal transplantation, who presented with metastatic colon cancer 5 years after transplantation.

**Case presentation:**

A 66-year-old Caucasian male patient, with hypertension, type 2 diabetes mellitus, paroxysmal atrial fibrillation, and renal failure underwent successful kidney transplantation in 2013. In April 2018, the adenocarcinoma of the sigmoid colon was found, and surgical resection was performed. The histological diagnosis was low-grade adenocarcinoma. Fluorodeoxyglucose positron emission tomography/computerized tomography scan showed a 2.5-cm metastasis in the VIIth segment of the liver and a metastatic paraaortical lymph node on the left. The clinical diagnosis was, therefore, metastatic (stage IV) sigmoid colon cancer (AJCC TNM system). The ongoing medications of the patient included immunosuppressive drugs and medication for his cardiovascular comorbidities. In July 2018, palliative cetuximab plus folinic acid–fluorouracil–irinotecan chemotherapeutic treatment was initiated, then cetuximab was substituted for panitumumab because of adverse events. In August 2018, the follow-up positron emission tomography/computerized tomography scan revealed stable disease. Because of side effects, the patient was unwilling to continue with the panitumumab plus folinic acid–fluorouracil–irinotecan treatment regimen. Therefore, the patient received 10× 5 Gy stereotactic body irradiation for his liver metastasis and mono-panitumumab therapy. By January 2019, the positron emission tomography/computerized tomography scan showed regression of the liver metastasis but a progression in the paraaortic lymph node. Therefore, 5× 8 Gy stereotactic irradiation was given to the paraaortic lesion. Meanwhile, the patient received altogether 16 cycles of panitumumab until June 2019, when complete remission was attained. In July 2019, the patient suffered a hemorrhagic stroke, probably due to his cardiovascular comorbidities, and died subsequently.

**Conclusions:**

Since information is scarce regarding oncological treatment of patients following organ transplantation, data about their oncological treatment is essential. To our knowledge, this is the first case report to describe the successful chemotherapy and targeted therapy supplemented with stereotactic radiotherapy of a posttransplant patient with metastatic colorectal cancer.

## Background

Cancer is the second leading cause of death worldwide, with colorectal cancer (CRC) being the second most common cause of all cancer-related deaths [1]. Previous analyses have shown that patients who underwent renal transplantation were at a greater risk of developing malignancies such as CRC than the general population [2–5]. Following organ transplantation, patients were three times more likely to develop cancer, with an overall cancer incidence of 1.9–18% [3, 4, 6]. Since risk of carcinoma has been shown to increase with long-term use of immunosuppressant drugs, the rise in cancer incidence following transplantation is most probably caused by the prolonged use of these agents [7, 8].

Because of advances in effective surgical techniques and immunosuppressive therapies, organ recipients live longer and the survival rates of grafts have also risen [2]. The increased survival rates of these patients and their higher CRC incidence rates indicate that a growing number of recipients will likely be requiring treatment for CRC.

Pharmacotherapy, including chemotherapy and targeted treatment, has emerged as a leading form of treatment for metastatic CRC [6]. This patient group, however, provides challenges for health care professionals regarding the selection of the appropriate oncological treatment. Patients take special medication after transplantation and tend to have various medical conditions that influence the feasibility and efficacy of oncological treatments. Yet, there is insufficient information and no clear guidelines regarding the recommended types of therapy for CRC patients following transplantation.

In the present case report, we describe the oncological treatment of a patient who had previously undergone renal transplantation and presented with metastatic colon cancer 5 years later.

## Case presentation

A 66-year-old Caucasian male patient, with a medical history of hypertension, type 2 diabetes mellitus (DM), paroxysmal atrial fibrillation, previous parathyroid adenoma, and renal failure of unknown origin underwent successful kidney transplantation in 2013. He did not smoke or consume alcohol and had no family history of CRC. In April 2018, he presented with abdominal discomfort at the Emergency Department. Gastroscopy showed ventricular erosions, while colonoscopy and abdominal computerized tomography (CT) scan revealed an adenocarcinoma of the sigmoid colon with minimal locoregional infiltration. Because of symptoms of bowel obstruction, urgent surgical resection was performed according to Hartmann. The histological diagnosis was low-grade adenocarcinoma, pT3 N1b (3/9+1TD), V1, Pn-, R0, N-K-Ras wild type. Fluorodeoxyglucose positron emission tomography (FDG-PET)/CT scan showed a 2.5-cm metastasis in the VIIth segment of the liver. A metastatic paraaortical lymph node on the left and raised the possibility of further smaller metastases in the liver, as well (Fig. 2a, f). Therefore, the clinical diagnosis of the patient was metastatic (stage IV) sigmoid colon cancer (AJCC TNM system). According to the multidisciplinary team (MDT), first-line palliative cetuximab plus folinic acid–fluorouracil–irinotecan (FOLFIRI) chemotherapeutic treatment was recommended.

**Fig. 1:**
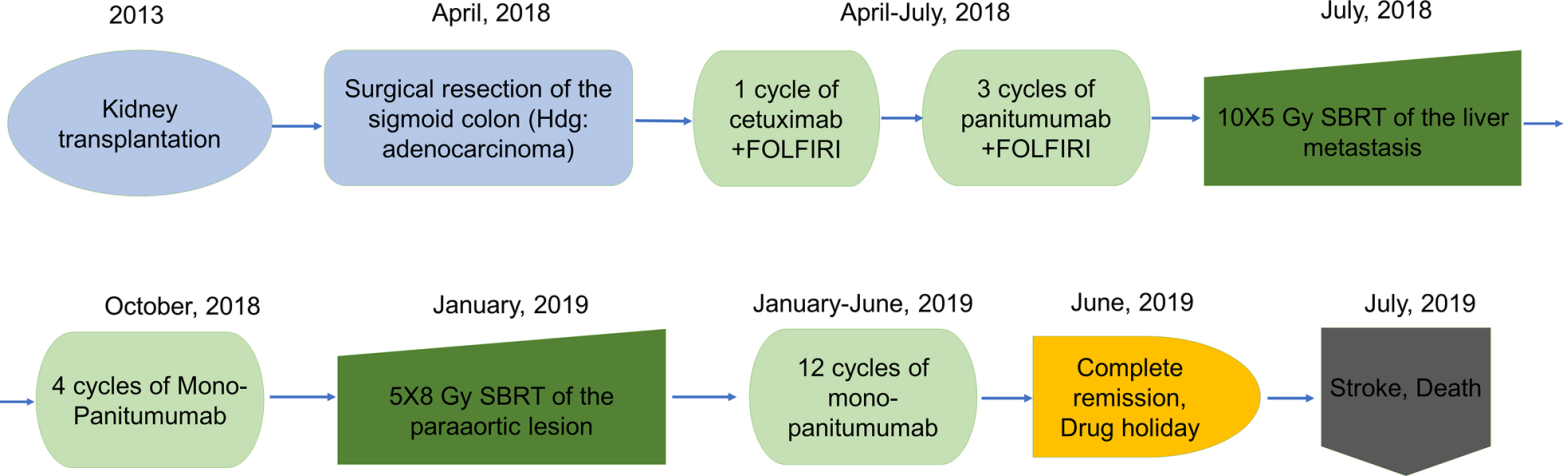
Flow chart of the main events of the patient’s illness and treatment

Figure 1 shows the main events of the patient’s illness and treatment.

The ongoing medications of the patient included immunosuppressive drugs: tacrolimus, methylprednisolone, and mycophenolate mofetil, as well as other drugs, losartan, pantoprazole, amlodipine, furosemide, potassium, calcitriol, atorvastatin, empagliflozin, dulaglutide, and rapid- and long-acting insulin. The patient’s body mass index was 30.9 kg/m^2^. His Eastern Cooperative Oncology Group (ECOG) performance was 1. Prior to initiation of the treatment, the patient’s routine laboratory tests were within normal limits and his blood pressure and blood sugar values were well controlled.

**Fig. 2:**
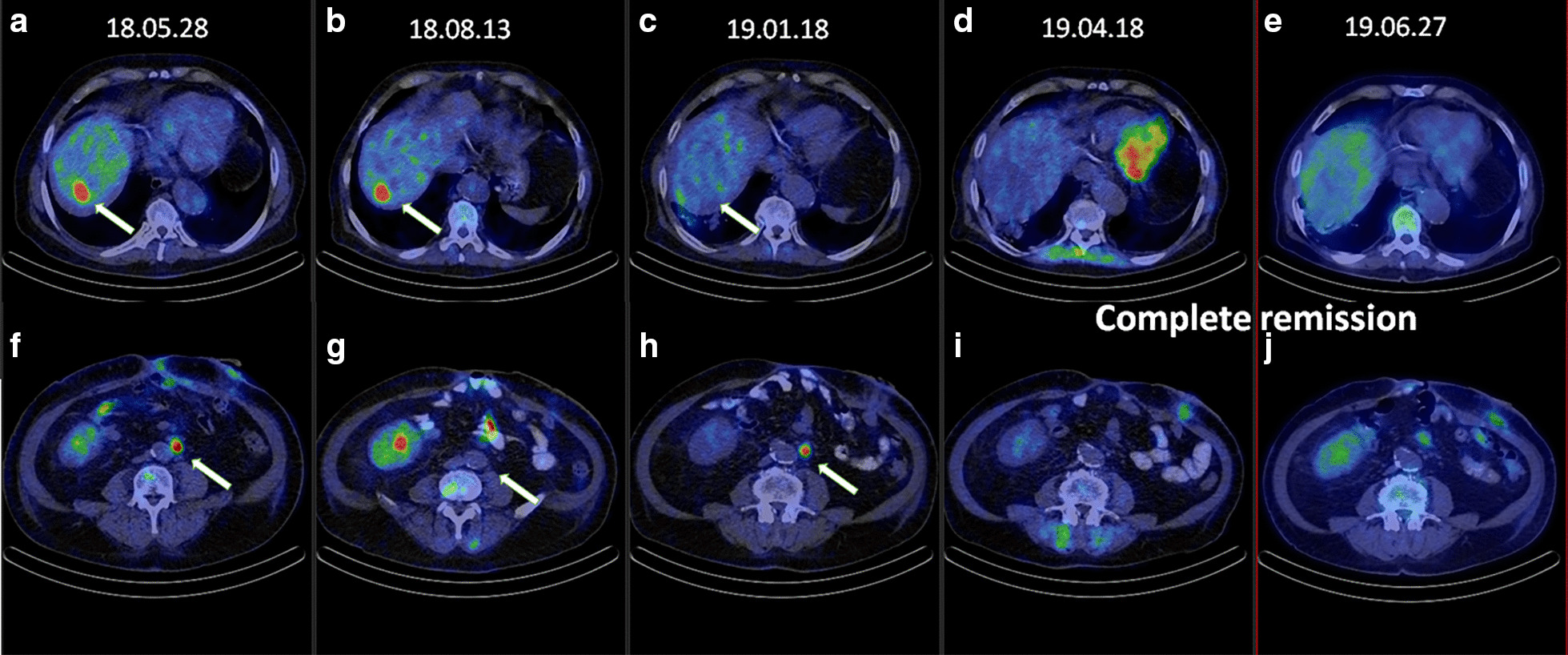
PET/CET scan images of the targets, the liver and the paraaortic (PAO) region in the different stages of treatment. **a**, **f** Image after surgery: visible metastases in the liver and PAO region (May, 2018). **b**, **g** Following targeted therapy plus FOLFIRI treatment: stable disease in the liver, the paraaortic lesion shows significant regression (August, 2018). **c**, **h** Following stereotactic body irradiation (SBRT), the lesion in the liver shows significant regression (January 2019). **d**, **i** Following SBRT of PAO region and concurrent mono-panitumumab treatment: signs of complete remission (April 2019). **e**, **j** June 2019: complete remission (June 2019)

Beginning July 2018, the patient received one cycle of first-line cetuximab-FOLFIRI therapy. Adverse events (AE) included fluctuations of blood sugar levels, diarrhea, and hypomagnesemia, which were treated adequately. To reduce the risk of further AE, cetuximab was substituted with panitumumab and the patient received panitumumab-FOLFIRI therapy through the second to fourth cycles of chemotherapy. The patient developed diarrhea, hypomagnesemia, and significant weight loss (5 kg) during treatment. In August 2018 the follow-up PET/CT scan revealed stable disease: regression of the left paraaortic lymph node, no progression in the size of the liver metastasis, and no local recurrence (Fig. 2b, g).

Because of the severe AE the patient was unwilling to continue with the panitumumab-FOLFIRI treatment regimen. Ablation with radiofrequency was not a possible option for treatment. Based on the MDT’s decision, multimodal therapy, including stereotactic radiotherapy of the liver metastasis was recommended. Accordingly, in September 2018 the patient received 10× 5 Gy stereotactic body irradiation (SBRT) for his liver metastasis, using the inspiration breath hold technique [9]. Since the patient did not consent to the continuation of the combination FOLFIRI treatment because of its side effects, monotherapy was initiated. Thus, the patient was given six cycles of mono-panitumumab therapy. AE, including skin rash and hypomagnesemia developed and were treated.

By January 2019, PET/CT scan showed regression of the liver metastasis but a progression in the paraaortic lymph node (Fig. 2c, d). Therefore, 5× 8 Gy stereotactic irradiation (SBRT) was given to the paraaortic lesion. The follow-up PET/CT scan revealed regression in both the size and metabolic activity of the paraaortic lymph node (Fig. 2d, i). Meanwhile, the patient continued to receive mono-panitumumab therapy and altogether received 16 cycles of panitumumab until June 2019. Complete remission was attained, since no macroscopic FDG-avid malignancy could be detected on the PET/CT scan at the end of June 2019 (Fig. 2e, j).

On 18 July 2019, the patient suffered a hemorrhagic stroke and became comatose. Despite neurosurgical intervention and intensive care, the patient died 10 days later.

## Discussion

To our knowledge, this is the first case report to describe the successful chemotherapy and targeted therapy supplemented with stereotactic radiotherapy of a posttransplant patient with metastatic CRC.

According to both earlier and recent analyses, renal transplant recipients had a significantly higher incidence of CRC [2, 3, 5] and a worse 5-year survival rate than the general population [10]. Although most patients were on average shown to be diagnosed one decade after transplantation, the patient in our case received his diagnosis of CRC much earlier, only 5 years after transplantation [2]. It must be noted, however, that our patient had type 2 DM and was obese, which are conditions that have been shown to be associated with an increased risk of CRC [2, 11].

At the time of diagnosis and throughout the treatment, our patient took immunosuppressive drugs. Posttransplant patients with advanced CRC sometimes do not receive adequate therapy due to physicians’ concern about harmful interactions between immunosuppressive and chemotherapeutic agents [12]. Our patient had metastatic CRC with one liver metastasis and one paraaortic lymph node involvement; therefore, cetuximab-FOLFIRI treatment was initiated.

FOLFIRI, a combination therapy consisting of 5-Fluorouracil (5-FU), leucovorin, irinotecan, and an epidermal growth factor (EGFR) inhibitor, cetuximab, has been shown to lead to an improvement in overall survival (OS) in metastatic CRC. The addition of cetuximab to FOLFIRI treatment has shown to increase OS from 15 to 18.5 months in right-sided tumors and from 21 to 28 months in left-sided tumors according to the CRYSTAL trial and from 15 to 18.3 months and 21.7 to 38.3 months in right- and left-sided tumors, respectively, according to the results of the FIRE-3 trial [13, 14]. The hypomagnesemia previously reported as an AE of cetuximab therapy [15, 16] was observed in our patient as well. Since our patient also developed fluctuations in blood sugar levels, biological treatment with the chimeric monoclonal antibody, cetuximab, was substituted with the fully human monoclonal antibody, panitumumab, in the subsequent cycles of FOLFIRI plus targeted therapy treatment. Although the follow-up PET/CT scan showed stable disease after four cycles of FOLFIRI plus targeted therapy, our patient could not continue the regimen because of intolerance (diarrhea and resulting significant weight loss) of the irinotecan-based chemotherapy. Therefore, the SBRT of the liver metastasis was carried out followed by subsequent cycles of panitumumab and irradiation of the paraaortic lymph node. Since systemic chemotherapy has been shown to improve patient survival in stage IV CRC, the relevance of the successful local treatment of oligometastases has also increased [17]. SBRT treatment of oligometastatic disease, characterized by an excellent safety profile, can efficiently complement systemic therapy, as was found in our patient’s case. The decision was in line with the current guidelines (NCCN Guidelines, colon cancer, Version 4.220). The associated adverse effects of panitumumab, skin rash, and decreased magnesium levels [18] developed in our patient’s case but were tolerable and could be treated adequately. Sixteen cycles of panitumumab as mono-treatment were successfully given, and follow-up was performed using PET/CT scans at regular intervals.

Thirteen months after diagnosis of metastatic CRC in our posttransplant patient, we were able to achieve complete remission. The patient subsequently died from a hemorrhagic stroke. The cause of his death was considered to be the result of his cardiovascular comorbidities and not due to the oncological treatment he received.

## Conclusions

Based on a growing number of studies, it appears crucial that recommendations for CRC screening be developed and implemented for posttransplant patients. Likewise, it is of considerable importance that these patients receive adequate treatment for CRC. Since information and guidelines are lacking regarding the treatment of patients after transplantation, data about their oncological treatment are essential. Our case report is valuable since we described the case of a metastatic CRC patient with renal transplant, where the use of chemotherapy was limited yet complete remission could be attained by using biological (targeted) treatment and radiotherapy.

## Data Availability

The datasets used and/or analyzed during the current study are available from the corresponding author on reasonable request.

## Abbreviations

CRC: Colorectal cancer
2 T DM: Type 2 diabetes mellitus
MDT: Multidisciplinary team
ECOG: Eastern Cooperative Oncology Group
AE: Adverse events
SBRT: Stereotactic body irradiation
5-FU: 5-Fluorouracil
EGFR: Epidermal growth factor
OS: Overall survival
